# A Novel Multi-Agent Model for Robustness with Component Failure and Malware Propagation in Wireless Sensor Networks

**DOI:** 10.3390/s21144873

**Published:** 2021-07-17

**Authors:** Biao Xu, Minyan Lu, Hong Zhang, Cong Pan

**Affiliations:** 1 The Key Laboratory on Reliability and Environmental Engineering Technology, Beihang University, Beijing 140191, China; lmy@buaa.edu.cn (M.L.); zh@buaa.edu.cn (H.Z.); cong_pan@buaa.edu.cn (C.P.); 2 School of Reliability and Systems Engineering, Beihang University, Beijing 140191, China

**Keywords:** WSN, cross-domain, agent-based model, malware, mathematical model, robustness, security

## Abstract

A wireless sensor network (WSN) is a group of sensors connected with a wireless communications infrastructure designed to monitor and send collected data to the primary server. The WSN is the cornerstone of the Internet of Things (IoT) and Industry 4.0. Robustness is an essential characteristic of WSN that enables reliable functionalities to end customers. However, existing approaches primarily focus on component reliability and malware propagation, while the robustness and security of cascading failures between the physical domain and the information domain are usually ignored. This paper proposes a cross-domain agent-based model to analyze the connectivity robustness of a system in the malware propagation process. The agent characteristics and transition rules are also described in detail. To verify the practicality of the model, three scenarios based on different network topologies are proposed. Finally, the robustness of the scenarios and the topologies are discussed.

## 1. Introduction

As a critical driver of the social evolution progress, the Internet has significantly transformed the way things communicate with each other. The Internet of Things (IoT) aims to promote this stride further to seamlessly connect people and various things, transforming society toward becoming intelligent, convenient, and efficient (ICE) with potentially excessive economic and environmental profits [1,2]. At the bottom layer of the IoT, smart, low-power, and micro-sensor devices are typically deployed to measure the physical conditions of the object or environment being monitored. These sensor devices are typically networked through wireless mediums, forming a wireless sensor network (WSN).

Due to some IoT application domains’ safety-critical or mission-critical features, it is compulsory that the WSN operate robustly throughout the intended mission time and mission environment. In other words, robustness is one of the crucial requirements for the adoption of the WSN in critical applications [3,4,5,6,7]. Malfunctions of WSN devices, failing to capture data, network outage, data corruption, or loss may cause catastrophic effects, such as mission failure, financial loss, and harm to people and environments.

For a more robust WSN, considerable research efforts have been disbursed in modeling and designing the reliability of WSN in the past decades. These studies are distributed at different levels, including the component level, path level, and system level [8].

The component-level reliability models aim to produce a realistic estimation of reliability or related attributes of nodes or links. Wang et al. [9] modeled the battery-powered sensor node (BPSN) reliability as the battery component, while Deif et al. [10] evaluated the BPSN reliability by considering the failures and dependencies of its four major constituent components. To make WSN resilient to sensor node failures, refs [11,12] studied several reliability designs using hot or cold standby sparing techniques. As for the path level, refs [13,14,15] analyzed the connectivity-based reliability of an end-to-end path (selected using a specific routing algorithm). The system-level WSN reliability has been defined and modeled based on the function performed. Researchers focus on the information transfer from sink node to sensor node [16,17,18] and/or the opposite direction [19,20,21]. Although the levels are different, it is undoubted that these component parameter estimations are crucial foundations of the reliability analysis at the path level and system level of WSN. In addition, higher network connectivity usually leads to higher resistance to node failure because it can provide multiple alternative connectivity paths.

Recently, increasing attention has been paid to the security of WSN. This trend has triggered our consideration of the security of WSN networks. According to current WSN reliability research, the information domain (for example, software failure and malware-related failure) is seldom considered in the reliability modeling of sensor nodes. To the best of our knowledge, no modeling work combines the propagation of malware and the impact of malware on the reliability of sensor nodes. WSN with high connectivity robustness needs a high average degree network topology, which will also accelerate the spread of malware, which may eventually reduce the connectivity robustness of WSN. If we only consider the component reliability or malware propagation factors to select the WSN network topology with higher connectivity reliability, then the results of selection may be quite different.

Consequently, in this work, an agent-based model (ABM) is proposed to analyze the malware propagation in WSN together with component reliability. We identify the individual physical-domain and information-domain characteristics of the agents involved, as well as the agent–agent and agent–environment interactions. The ABM presented in this research will use mathematical epidemiology to determine the states of agents in each slice of time and choose the maximally connected component (MCC) as the measure to evaluate the connectivity robustness of WSN. The SEIRS-F ABM proposed in this study considers system characteristics such as the reliability of sensor nodes, malware, topology, environment, and maintenance. The main contributions of this work are listed as follows.

- We propose a cross-domain ABM. The model integrates the physical domain (component failure) and information domain (malware propagation) and can model the cascading failures between the physical and information domains.
- We consider a cross-domain description and classification of agents, which includes not only sensors but also other aspects such as malware, network topology, environment, and maintenance.
- We carry out a series of simulations based on NetLogo with different scenarios and topologies. Scenarios include military, industry, and Smart Home scenarios. Topologies include star, lattice-2d, random, and complex networks. The real-time display of the discrete states of the node and the MCC at each step of time t is shown.
- We compare the robustness of each scenario and topology based on the simulation results. We also compared with an existing model; the results show that there is a significant difference in the robustness of the model considering malware propagation compared to the control model. Noting that malware is already one of the important factors affecting WSN reliability, our model is more realistic.

The rest of the paper is organized as follows. Section 2 gives an overview of recent research in the field of component reliability and mathematical models that have been proposed to model malware propagation in WSN. In Section 3, the SEIRS-F agent-based model to simulate node failure and malware propagation in WSN is presented. For simulations, the model parameters obtained according to the application scenarios are given in Section 4. Section 5 discusses the simulation results. Finally, in Section 6, the conclusions are presented.

## 2. Related Work

### 2.1. Component Reliability Models

A WSN is a collection of a large number of sensor nodes that are deployed over an area or inside a target that needs to be detected, monitored, or tracked [22]. These nodes self-organize into a cooperative network [23], communicate in an ad hoc manner, and transmit sensor measurements to the end-user.

A typical sensor node contains four major components: a sensing component for data acquisition, a processing component for local data computing, a radio or communication component for data transformation, and a power supply component [24,25]. Other components, such as the locating component or actuator (for sensor adjustment and movement), may also be part of sensor nodes in specific applications.

Several issues can affect the reliability of WSN by degrading its functionality in terms of coverage and/or connectivity. These concerns are mainly power supply failure, subsystem failure, and software failure. These issues can be summarized as follows:

Power supply failure: most of the industrial and commercial SNs are battery-powered. The latest development of battery manufacturing has recently introduced high durability batteries (e.g., lithium thionyl chloride batteries) for SNs that can be used for years under certain conditions. Although these batteries can support the operation of SNs for an extended period, premature battery failures may still occur in practice. The failure can be attributed to various reasons, such as the deployment of SNs in harsh environmental conditions (e.g., extreme temperatures or rain), incorrect handling, or random failures caused by defective hardware.

Subsystem failure: SNs are affected by random hardware failure for two main reasons. First, most commercial SNs are cost-sensitive, which means they are not always made up of the highest quality subsystems. Second, SNs are often subjected to adverse environmental conditions, which will affect the regular operation of their components [26].

Software failure: SNs are prone to random permanent software failures, rendering them inactive, i.e., unable to sense or communicate [22].

The power supply sources divide the sensor nodes into two categories, battery-powered sensor node (BPSN) and energy-harvesting sensor node (EHSN), that can convert ambient energy into electrical energy [27]. Wang et al. [9] modeled the reliability of a BPSN as its battery lifetime. Because the limited battery lifetime essentially determines the lifetime of BPSN in many practical scenarios, it is impossible to charge or replace the sensor battery during the mission [28]. Zonouz et al. [13] modeled both BPSN and EHSN as multi-subsystem components and evaluated their reliabilities based on the reliability of each subsystem’s energy flow and reliability. Deif et al. [10] assessed the BPSN reliability by considering failures and dependencies of its four major components.

The fault-tolerant design of nodes is also critical for reliability. To make WSN resilient to sensor node failures, Xing et al. [11] investigated and compared several reliability designs using hot or cold standby sparing techniques. Xing and Shrestha [12] considered the hot standby sparing design to achieve a highly reliable sink node subsystem.

Path level reliability design is usually realized by the multi-path routing protocol [14,15], and link reliability/path reliability is considered in routing algorithm design. Routing algorithms and protocols play a significant role in the reliability of the communication/networking layer. The routing algorithm used in the protocol determines the information transmission path from the source node to the destination node. When a node or link fails on the selected path, a reliable routing protocol is responsible for detecting the failure and finding an alternative path to complete the required information transmission.

System-level reliability pays attention to the execution of actual functions, usually related to connectivity [29]. Researchers study the information transfer from sink node to sensor node [16,17,18] and/or the opposite direction [19,20,21].

Although the levels are different, it is undoubted that these component parameter estimations are crucial foundations of the reliability analysis at the path-level and system-level of WSN. In addition, higher network connectivity usually leads to higher resistance to node failure because it can provide multiple alternative connectivity paths.

### 2.2. Malware Propagation Models

Modeling the propagation of malware in computer networks has a history of decades. These studies have established the foundation for malware propagation modeling in WSN. The mathematical models proposed to study the malware propagation in WSN can be global or individual. In this section, the two types of models are briefly described. In addition, these models have been classified according to their compartmental model types (e.g., continuous or discrete, deterministic or stochastic) and mathematical models used (e.g., partial differential equations (PDE), ordinary differential equations (ODE), cellular automata (CA), and Markov chains).

Starting with the global model, Zhu et al. [30] described an SIR model, which considers the discrete delay and can effectively predict the dynamic behavior and spatial distribution of malware propagation in mobile WSN. The proposal is a continuous and deterministic model. Shen et al. [31] present a heterogeneous susceptible–insidious–infectious–recovered–dysfunctional (SNIRD) model on WSN. The model included the N state as the infected sensor that has not been distinguished by the intrusion detection system (IDS). The D state symbolized the node that failed due to malware demolition, power exhaustion, or physical damage. This model is discrete and stochastic. The two models are both based on PDE.

Feng et al. [32] proposed an improved SIRS model to describe the energy consumption during worm propagation and different node distribution density. This model is continuous and deterministic. Liu et al. [33] studied the malware spreading on WANETs. Based on the classical SI epidemic propagation model, the propagation rates of two malware propagation modes and two different network modes are studied. It is a discrete and deterministic model. Acarali et al. [34] proposes an IoT–SIS botnet propagation model based on IoT sensor networks. The impact of IoT-specific characteristics such as limited processing power, energy restrictions, and node density on the formation of a botnet was analyzed. It is a discrete and deterministic model. The three models are based on ODE.

Shen et al. [35] presented a heterogeneous discrete-time SIS model. They developed a non-cooperative non-zero-sum game to describe heterogeneous WSN and malware interaction to predict malware’s infection behavior. Wu et al. [36] proposed a SIRD model to evaluate malware propagation on the narrowband Internet of Things (NBIOT). The nodes’ availability based on the distribution of heterogeneous nodes and vulnerability was analyzed. These two models are based on Markov chains. Therefore, they are discrete, stochastic models.

Subsequently, the specific network characteristics have also been taken into account in individual models.

Wang et al. [37] introduced a model that follows the state transition scheme of a typical SI infection model. Nevertheless, they could compute the prior probability of each sensor being infected by the worm using several iterative equations of individual security states. The Markov chains model is individual, discrete, and stochastic.

Del Rey et al. [38] proposed an improved individual-based model, which used characteristics of three types of nodes and complex topology. The states of the model were susceptible, infected, recovered, damaged, and out-of-order. Batista et al. [39] presented an SEIR model to simulate the spread of computer viruses on a computer network. In this model, the parameters considered are related to the life cycle of the computer virus, the countermeasures implemented on the host, and the user’s behavior. Wang et al. [40] proposed a SIRD model based on two-dimensional 2D cellular automata CA. This model considered three aspects (infection, immunity, and mortality rates) in two different types of nodes (cluster-head and terminal nodes) to analyze malware propagation in WSN. In addition, a multi-player evolutionary game model was established to find the optimal evolutionary and stable strategy. The previous three studies used cellular automata, and the models were individual, discrete, and deterministic.

The ABM paradigm has also been considered for designing models to simulate biological agent spread [13,14,15] and malware propagation [41,42].

The novel developed ABM paradigm model allows each autonomous individual to have its characteristics and action rules and establish individual–individual or individual–environment interactions. The features often considered in related research are the types of sensors and malware. Some recent studies included topology and environment. In addition to these particularities, the model also includes other sensor characteristics, such as computational capacity, information communication capacity, duty cycle, and data acquisition method. Some other studies introduced human and external, and computational devices as agents to influence the spread of malware.

## 3. SEIRS-F Agent-Based Model

The SEIRS-F (i.e., susceptible, exposed, infected, recovered, and failed) agent-based model proposed in this work is an individual, discrete, and stochastic model. This model has allowed analyzing the component failure behavior and the malware behavior from a cross-domain perspective by integrating new elements that will enable the adjustment of the characteristics of the model to more realistic interactions between component and malware. Therefore, sensors, malware, network topology, environment, and maintenance have been defined as agents. Additionally, transition rules have been adjusted with characteristics of different agents and behaviors of the WSN in the environment.

Additionally, the behavior and characteristics of an agent can be evaluated individually in a slice of time t. Finally, this model uses the advantages of agent-based models for cross-domain dependency in wireless sensor networks.

**Figure 1:**
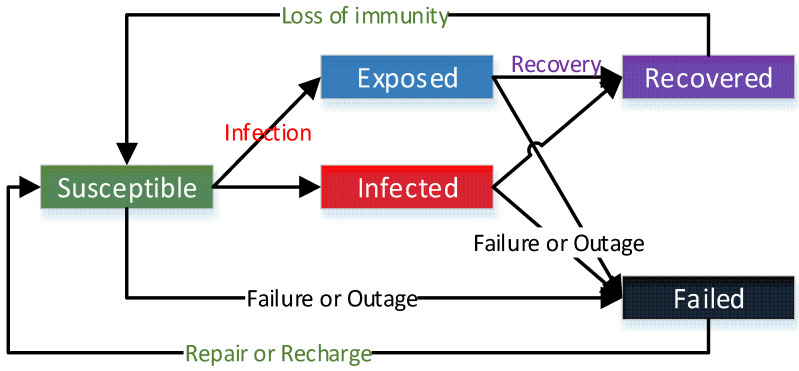
Scheme of the SEIRS-F model.

In the proposed SEIRS-F model, the sensor nodes adopt, in each slice of time t, one of the following states (see Figure 1):

Susceptible: the sensor is working correctly without subsystem failure and has not been infected by malware, but it has the characteristics to be failed or infected.

Exposed: the sensor is reached by malware but cannot transmit malware to the neighbor sensor due to the characteristics of the sensor. Whether the sensor works properly depends on the type of malware.

Infected: the sensor infected by malware. The infected sensor obtains the ability to transmit malware to its neighbors. Whether the sensor works appropriately depends on the type of malware.

Recovered: the sensor acquires temporal resistance to malware when it has successfully removed malware or installed security fixes.

Failed: the sensor that dies because of malicious physical damage, subsystem failure, battery life out, or the quick discharge when infected by malware.

The population of nodes is supposed to remain constant, consequently: $S\left(t\right)+E\left(t\right)+I\left(t\right)+R\left(t\right)+F\left(t\right)=N$ at each step of time t. For a given time t, N is the total number of agents, $S\left(t\right)$ are the susceptible agents, $E\left(t\right)$ stands for the number of exposed agents, $I\left(t\right)$ represents the number of agents in the infected state, $R\left(t\right)$ are the recovered agents, and finally, $F\left(t\right)$ denotes the agents in the failed state.

Agents concern the meaning of autonomy and interact, collaborate, coordinate, and negotiate with both each other and the environment, based on the transition rules. In the model proposed in this work, the agents and the characteristics will be defined in Section 3.1. In Section 3.2, these transition rules will be detailed according to agents’ behavior, which is established by rules that define the agents’ response to other agents and the environment.

### 3.1. Agents and Environmental Characteristics

Sensors and some other environmental factors compose the SEIRS-F agent-based model—sensor nodes, malware, network topology, environment, and maintenance resource. These factors have been selected after analyzing the different characteristics and environments that may affect the operation of the wireless sensor network. The main characteristics considered for each factor are as follows: (1) importance to the component failure process and maintenance process and (2) contribution to the malware propagation process.

The factors with their specific characteristics and corresponding values are described in Table 1.

Sensors can collect data from the environment, which is the main element in WSN. Based on the consideration of reliability and security, we will consider the following characteristics of sensors:

- Reliability state of sensor: the sensor has two states, i.e., the normal state and the failure state. The sensor can achieve the expected function such as collection, processing, and transmission in the normal state. While in the failure state, one or all of these functions cannot be accomplished.
- Reliability level: has been divided into a high level, medium level, and low level, corresponding to the sensor nodes whose reliability level is from high to low.
- Security level: has been classified into high level when the trusted security methods are used, medium-level if it uses basic means, and low level if it does not have any security designs.
- Transmission capability: Some nodes are limited by software and hardware function and cannot spread malware to their neighbors.
- Battery power: essentially determines the lifetime and thus the reliability of the sensor node [25,43]. The initial battery power is divided into three levels, from low to high.

Malware is any software intentionally designed to cause damage to a computer, server, client, or computer network. The following two characteristics of malware are considered in our model:

- Infection intensity is the attack strength of malware; higher infection intensity has a greater possibility of infecting the node’s neighbors. Infection intensity may vary between very high and very low.
- Target: malware is designed for different targets, some of which hinder the function of the software; some could increase energy consumption and accelerate the consumption of battery lifetime; others can even damage the hardware. The target can be software function, subsystem hardware, or battery power.

Network topology: refers to the structure of the network. We will consider one characteristic of this agent:

- Type of topologies: star topology, lattice-2d topology, and random topology.

Environment: differs regarding the type and application of the WSN. In this work, we divided the environment by the risk of physical attack and the risk of malware attack, as follows:

- Risk of physical attack: sensors are deployed in hostile environments in a military surveillance application, which means high risk. Some attackers may attack the physical devices and information functions of the industrial infrastructure at the same time to achieve a higher success rate, which leads to medium risk. Sensors in other application scenarios are usually deployed in a friendly environment with close attention, and the risk of intentional physical attack is shallow, which is generally just accidental damage.
- Risk of malware attack: Military environment corresponds to high risk, medium risk when the environment is industrial, and low risk when the environment is daily activities and Smart Home.

Maintenance: this is related to the maintenance resources, behaviors, and cycle. The connectivity robustness of WSN is maintained by restoring the function of nodes after they suffer from physical or information shock. The two characteristics considered here are:

- Resource: The available maintenance resources, including materials and workforce, are not unlimited. The amount of resources is divided into a high level, medium level, and low level.
- Maintenance behavior: in some environments, it is inconvenient or even impossible to recharge or repair the sensor nodes for WSN in hostile environments such as military surveillance applications. The failed nodes can only be replaced by re-dropping nodes regularly. In other scenarios, the node can be repaired or charged, but it also costs human resources.
- Maintenance cycle: each can reset the remaining maintenance resources. There is an upper limit for one-time input of resources, and resources can be recovered over time. Due to the work cycle of humans and equipment, the recovery of maintenance resources is designed to be periodic.

### 3.2. Transition Rules

The transition rules of the SEIRS-F model define the conditions that a sensor $x_{i}$ must be satisfied to transform from one state to another in a step of time t, where the state of $x_{i}\in \left\{S,E,I,R,F\right\}$. These rules are designed based on previously defined agent characteristics.

Transition rules define the interaction patterns between sensors and their environment.

#### 3.2.1. Susceptible to Infected

A sensor transforms from a susceptible to an infected state when it is compromised by malware during an attack. The probability of a node changing from susceptible to infected depends on five characteristics: the reliability state of the sensor, as a failed node will not be infected by malware; transmission capability, as some nodes are limited by hardware and software resources and cannot spread malware; the security level of the sensor and the infection intensity of malware are two opposite characteristics, which constitute the probability of the sensor being infected by the malware; and there is also the risk of malware attack, which affects the number of initially infected nodes.

The explicit expression is the following:

(1) $$P\left[x_{i}\left(0\right)=S\left(0\right)\to x_{i}\left(0\right)=I\left(0\right)\right]=\beta \left(X_{10}\right)\;when\;X_{1}=normal\;state\;\mathrm{AND}\;X_{4}=true$$

(2) $$P\left[x_{i}\left(t\right)=S\left(t\right)\to x_{i}\left(t+1\right)=I\left(t+1\right)\right]=\sigma \left(X_{3},X_{6}\right)\;when\;X_{1}=normal\;state\;\mathrm{AND}\;X_{4}=true$$

where $X_{1}$ represents the reliability state of the sensor, $X_{4}$ indicates the transmission capability, $X_{3}$ means the security level of the sensor, $X_{6}$ reflects the infection intensity of the malware, and $X_{10}$ considers the risk of malware attack.

#### 3.2.2. Susceptible to Exposed

A sensor moves from a susceptible to an exposed state when it has been captured by malware but does not have the processing capacity to disseminate the malware to neighboring nodes. The probability of a node switch from susceptible to exposed depends on the same five characteristics as the previous transition rule; merely one of the features has changed.

That is:

(3) $$P\left[x_{i}\left(0\right)=S\left(0\right)\to x_{i}\left(0\right)=E\left(0\right)\right]=\beta \left(X_{10}\right)\;when\;X_{1}=normal\;state\;\mathrm{AND}\;X_{4}=false$$

(4) $$P\left[x_{i}\left(t\right)=S\left(t\right)\to x_{i}\left(t+1\right)=E\left(t+1\right)\right]=\sigma \left(X_{3},X_{6}\right)\;when\;X_{1}=normal\;state\;\mathrm{AND}\;X_{4}=false$$

where $X_{1}$ represents the reliability state of the sensor, $X_{4}$ indicates the transmission capability, $X_{3}$ means the security level of the sensor, $X_{6}$ reflects the infection intensity of the malware, and $X_{10}$ considers the risk of malware attack.

#### 3.2.3. Susceptible to Failed

A sensor moves from a susceptible to a failed state when its subsystems (including power subsystem, sensing subsystem, processing subsystem, communication subsystem, and other specific subsystems) fail, or the software fails, or the battery power is exhausted. The probability of a node switch from susceptible to failed depends on four characteristics: the reliability state of the sensor, as a failed node will not fail again; the reliability level of the sensor, which contains the reliability level of hardware and software; battery power, which is the essential condition of sensor operation; and the risk of physical attack, as sensors deployed in hostile environments are highly likely to be physically damaged. This characteristic affects the number of sensors that are initially physically destroyed and fail.

That is:

(5) $$P\left[x_{i}\left(0\right)=S\left(0\right)\to x_{i}\left(0\right)=F\left(0\right)\right]=\delta \left(X_{9}\right)\;when\;X_{1}=normal\;state$$

(6) $$P\left[x_{i}\left(t\right)=S\left(t\right)\to x_{i}\left(t+1\right)=F\left(t+1\right)\right]=1\;when\;X_{1}=normal\;state\;\mathrm{AND}\;X_{5}\leq 0$$

(7) $$P\left[x_{i}\left(t\right)=S\left(t\right)\to x_{i}\left(t+1\right)=F\left(t+1\right)\right]=\lambda \left(X_{2}\right)\;when\;X_{1}=normal\;state\;\mathrm{AND}\;X_{5}>0$$

where $X_{1}$ represents the reliability state of the sensor, $X_{2}$ indicates the reliability level, $X_{5}$ means the remaining battery power of the sensor, and $X_{9}$ reflects the risk of physical attack, which affects the number of nodes that fail initially due to physical damage.

#### 3.2.4. Infected to Failed

Similar to the previous transition rule, a sensor moves from an infected to a failed state when its subsystems fail, the software fails, or the battery power is exhausted. However, a characteristic related to transition probability is added, which is the target. Different malware will cause different types of damage to the sensor. Some will directly attack the hardware and software, resulting in sensor failure. Some others could accelerate power consumption, eventually leading to power exhaustion and sensor failure.

It is supposed that:

(8) $$P\left[x_{i}\left(t\right)=I\left(t\right)\to x_{i}\left(t+1\right)=F\left(t+1\right)\right]=1\;when\;X_{1}=normal\;state\;\mathrm{AND}\;X_{5}\leq 0$$

(9) $$P\left[x_{i}\left(t\right)=I\left(t\right)\to x_{i}\left(t+1\right)=F\left(t+1\right)\right]=\lambda \left(X_{2}\right)\;when\;X_{1}=normal\;state\;\mathrm{AND}\;X_{5}>0\;AND\;X_{7}\;is\;not\;software\;function\;or\;subsystem\;hardware$$

(10) $$P\left[x_{i}\left(t\right)=I\left(t\right)\to x_{i}\left(t+1\right)=F\left(t+1\right)\right]=\lambda \left(X_{2},X_{7}\right)\;when\;X_{1}=normal\;state\;\mathrm{AND}\;X_{5}>0\;AND\;X_{7}\;is\;software\;function\;or\;subsystem\;hardware$$

where $X_{1}$ represents the reliability state of the sensor, $X_{2}$ indicates the reliability level, $X_{5}$ means the remaining battery power of the sensor, and $X_{7}$ reflects the target of the malware.

#### 3.2.5. Exposed to Failed

Analogous to the past transition rule, a sensor passes from an exposed to a failed state when its subsystems fail, the software fails, or the battery power is exhausted. The difference between the infected state and the exposed state is whether the node can spread malicious software, which does not affect the state transformation of the node itself.

As a consequence:

(11) $$P\left[x_{i}\left(t\right)=E\left(t\right)\to x_{i}\left(t+1\right)=F\left(t+1\right)\right]=1\;when\;X_{1}=normal\;state\;\mathrm{AND}\;X_{5}\leq 0$$

(12) $$P\left[x_{i}\left(t\right)=E\left(t\right)\to x_{i}\left(t+1\right)=F\left(t+1\right)\right]=\lambda \left(X_{2}\right)\;when\;X_{1}=normal\;state\;\mathrm{AND}\;X_{5}>0\;AND\;X_{7}\;is\;not\;software\;function\;or\;subsystem\;hardware$$

(13) $$P\left[x_{i}\left(t\right)=E\left(t\right)\to x_{i}\left(t+1\right)=F\left(t+1\right)\right]=\lambda \left(X_{2},X_{7}\right)\;when\;X_{1}=normal\;state\;\mathrm{AND}\;X_{5}>0\;AND\;X_{7}\;is\;software\;function\;or\;subsystem\;hardware$$

where $X_{1}$ represents the reliability state of the sensor, $X_{2}$ indicates the reliability level, $X_{5}$ means the remaining battery power of the sensor, and $X_{7}$ reflects the target of the malware.

#### 3.2.6. Infected to Recovered

A sensor shifts from an infected to a recovered state when the malware of the compromised node has been removed, and the node temporarily gains immunity to malware. The duration of temporary immunity relates to the security level of the sensor and the infection intensity of malware, which both decide the duration of immunization.

The probability of a node switch from infected to recovered depends on three characteristics. The security level of the sensor and the infection intensity of malware represent the technical strength of the defense and the attacker, respectively. The more notable the gap between the two, the easier/harder it is to remove malware from nodes and gain immunity. In the maintenance cycle, the premise of installing security patches is that malware infection is found during maintenance. In the simulation, the maintenance cycle decreases with time. When it is equal to 0, the maintenance is executed, and the maintenance cycle resets.

It is defined as follows:

(14) $$P\left[x_{i}\left(t\right)=I\left(t\right)\to x_{i}\left(t+1\right)=R\left(t+1\right)\right]=\gamma \left(X_{3},X_{6}\right)\;when\;X_{13}=0$$

where $X_{3}$ represents the security level of the sensor, $X_{6}$ indicates the infection intensity of the malware, and $X_{13}$ means the maintenance cycle.

#### 3.2.7. Exposed to Recovered

Like the previous transition rule, the sensor moves from an exposed to a recovered state when the malware has been detected and removed, and the node temporarily gains immunity to malware. Both transition rules are mainly the same.

As a consequence:

(15) $$P\left[x_{i}\left(t\right)=E\left(t\right)\to x_{i}\left(t+1\right)=R\left(t+1\right)\right]=\gamma \left(X_{3},X_{6}\right)\;when\;X_{13}=0$$

where $X_{3}$ represents the security level of the sensor, $X_{6}$ indicates the infection intensity of the malware, and $X_{13}$ means the maintenance cycle.

#### 3.2.8. Recovered to Susceptible

A sensor shifts from a recovered to a susceptible state when it loses its temporary immunity. While the node obtains security repair, the attacker will also update the malicious code—the node cannot get immunity once and for all.

Then:

(16) $$P\left[x_{i}\left(t\right)=R\left(t\right)\to x_{i}\left(t+1\right)=S\left(t+1\right)\right]=1\;when\;immunity\;duration=0$$

where $X_{1}$ represents the reliability state of the sensor, $X_{2}$ indicates the reliability level, $X_{5}$ means the remaining battery power of the sensor, and $X_{7}$ reflects the target of the malware.

#### 3.2.9. Failed to Susceptible

A sensor alternates from failed to susceptible state when the sensor failure has been repaired. The failure may be caused by subsystem hardware failure, software failure, and power exhaustion. Various deployment environments and failure reasons affect the choice of maintenance behavior. For example, in military applications, sensors deployed in a hostile environment are challenging to repair or charge. Hence, they often choose to put in new sensors to replace the failed sensors directly.

Furthermore, different maintenance behaviors correspond to different maintenance resource consumption, which will affect the remaining maintenance resources. The probability of a node switch from failed to susceptible depends on three characteristics: the resource of maintenance, as the maintenance of failed sensor node consumes a certain amount of resources; maintenance behavior, which corresponds to different resource consumption; and the maintenance cycle, as each cycle resets the remaining maintenance resources.

It is supposed that:

(17) $$P\left[x_{i}\left(t\right)=F\left(t\right)\to x_{i}\left(t+1\right)=S\left(t+1\right)\right]=1\;when\;X_{11}>0\;\mathrm{AND}\;X_{13}=0$$

where $X_{11}$ represents the remaining maintenance resources, $X_{13}$ indicates the maintenance cycle, $X_{12}$ is implied in the change of $X_{11}$ and different $X_{12}$ corresponds to different consumption quantities of $X_{11}$.

## 4. Simulation

Some software supports ABM modeling and simulation, including free open source software and paid commercial software. Each solution brings valid characters for different domains that can be studied. Abar et al. [44] summarized the tools used to model and simulate these models and their application areas and analyzed the ease of model development and computational modeling ability; another critical feature is the scalability of the models.

The simulation of the SEIRS-F model has been developed in NetLogo [45]. The framework has been chosen according to the number of supported nodes, implementation area, programming convenience, and result visualization. In this case, a network of 140 nodes, a personal computer as the implementation area, NetLogo’s language as the programming language, graphics generation, and real-time results visualization have been easily obtained for the selected simulation tool.

**Figure 2:**
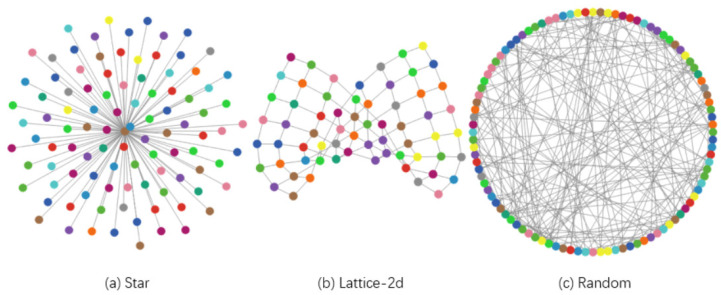
View of network topologies.

The model’s simulation is implemented with 140 nodes; each node corresponds to a sensor agent, and three network topologies have been defined in each scenario (see Figure 2): star, lattice-2d (basically, a grid), and random (generating algorithm uses the G (n, p) variant of the Erdős–Rényi model [46]). We choose the maximally connected component (MCC) as the measure to evaluate the connectivity robustness of WSN.

Next, different scenarios are described, and the simulation results under the combination of scenario and topology are given. These scenarios include military, industry, and Smart Home, which are typical application scenarios for WSNs. They have significant differences in the reliability and security of sensor nodes, malware infection intensity, the number of maintenance resources invested, and how they are invested due to different stakeholder considerations. We have additionally added simulation experiments on complex networks to explore the MCC performance of more network topologies. See Appendix A for parameter settings for all these simulations.

### 4.1. Scenario 1

The environment is the military surveillance application in this scenario.

The reliability and security of military sensors are highly required. To maintain long-term work, the battery power is large.

The infection intensity in terms of the attacker’s technical level is high, and the attackers may choose multiple targets.

Nodes are usually deployed in malicious areas, and the risks of physical attacks and malware attacks are high.

Due to the private nature of the mission and the inaccessibility of the hostile territory, it may not be possible to repair or recharge the sensors during the task; the maintenance team chose to replace the failed sensor with the intact one. The maintenance resources and maintenance cycle are high (see Table A1 and Table A2).

**Figure 3:**
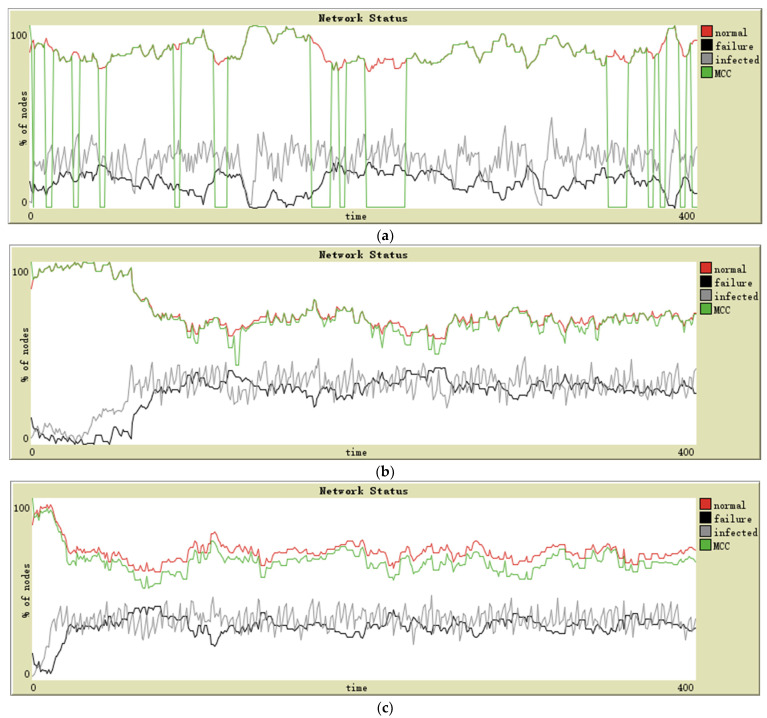
Simulation on military surveillance application in (**a**) star topology, (**b**) lattice-2d topology, and (**c**) random topology.

The three topologies have been defined with the same feathers: 140 sensor nodes and ten random intransmissible nodes. The connection probability of each node in a random topology is 0.03. These simulation designs have resulted in the following graphs for star (see Figure 3a), lattice-2d (see Figure 3b), and random (see Figure 3c) topologies. The evolution of the MCC in each topology is observed. In order to distinguish the MCC curve from the normal curve in the image, we slightly reduce the values of each point on the MCC curve. The following figures are processed in the same way.

### 4.2. Scenario 2

The setting for this scenario is the industry.

Industrial sensors have high-reliability requirements and have a general level of security. A peaceful deployment environment allows the sensor to be charged, so the battery capacity is suitable.

Some malicious attackers will implant computer viruses into the enterprise’s industrial facilities for ransom. The organized hacker groups are skillful in technical means. They usually choose the software function as the target and do not seek to obliterate the WSN—that is against the purpose of blackmail.

To improve the attack’s success rate, it is also feasible to confuse the maintenance team with simultaneous physical destruction. The risk of a physical attack is medium, and the risk of a malware attack is high.

The inspection and maintenance of industrial sensors is a routine work of the maintenance team, and the maintenance resource and maintenance cycle are medium (see Table A4 and Table A5).

**Figure 4:**
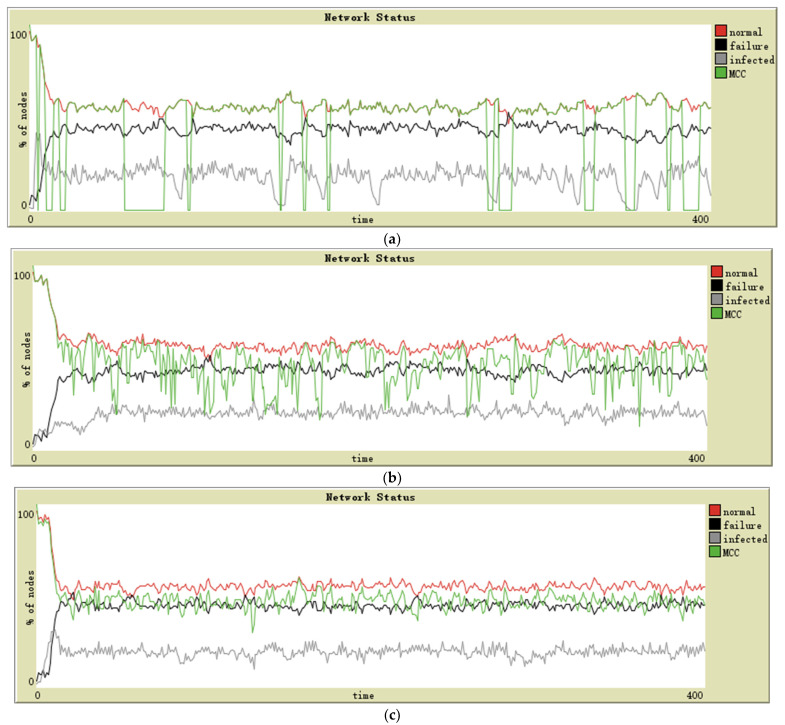
Simulation on industry application in (**a**) star topology, (**b**) lattice-2d topology, and (**c**) random topology.

Configurations of the topologies are the following: 140 sensor nodes and ten random intransmissible nodes. The connection probability of each node in a random topology is 0.03. The simulation results are in the following graphs for star (see Figure 4a), lattice-2d (see Figure 4b), and random (see Figure 4c) topologies. The evolution of the MCC in each topology is observed.

### 4.3. Scenario 3

The last environment has been defined with daily activities and Smart Home.

Considering the cost, the reliability and security of Smart Home sensors are low. The charging of daily equipment is convenient, so the battery power is low.

Attacking individual users can only gain little for skilled attackers, and it is not convenient to obtain ransom money. This type of information attack is often large-scale, using less advanced malicious code, not specifically for someone. The infection intensity is low, and the target is software function.

The risk of physical attacks and malware attacks is low.

Problems with daily necessities can usually be found and maintained immediately. Maintenance resource and maintenance cycle are low (see Table A6 and Table A7).

**Figure 5:**
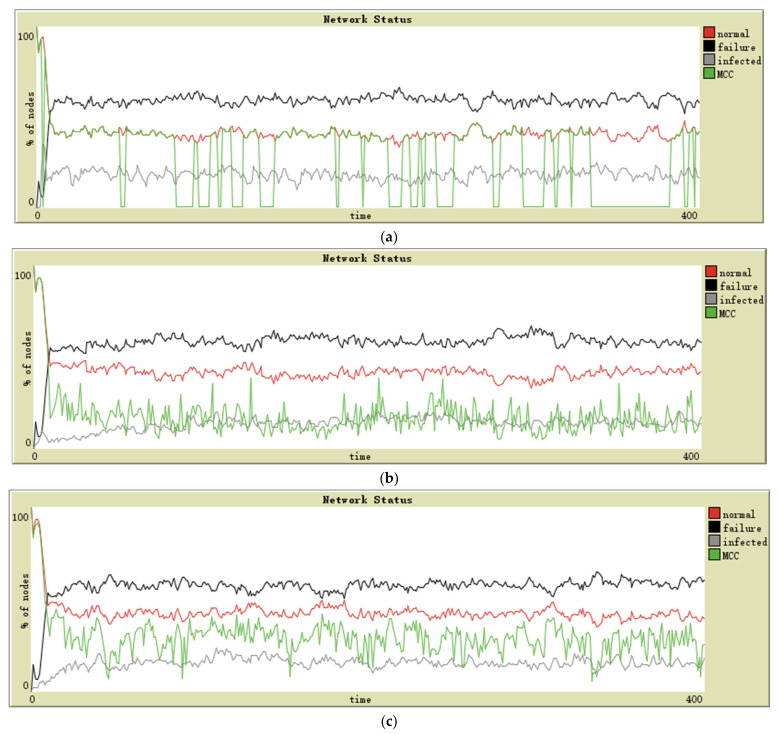
Simulation on Smart Home in (**a**) star topology, (**b**) lattice-2d topology, and (**c**) random topology.

The following topologies have been defined: 140 sensor nodes and ten random intransmissible nodes. The connection probability of each node in random topology is 0.03. Next, the results obtained in this scenario can be seen in (Figure 5a–c).

### 4.4. Complex Networks

To make a simulation comparison on a larger scale topology with complex network properties, we add simulation experiments of complex networks. Due to the large scale of complex networks and a substantial number of nodes and connections, the simulation speed is affected. Complex networks’ simulation has only been implemented in military applications, and the parameter design is the same as Scenario 1 (see Table A3).

**Figure 6:**
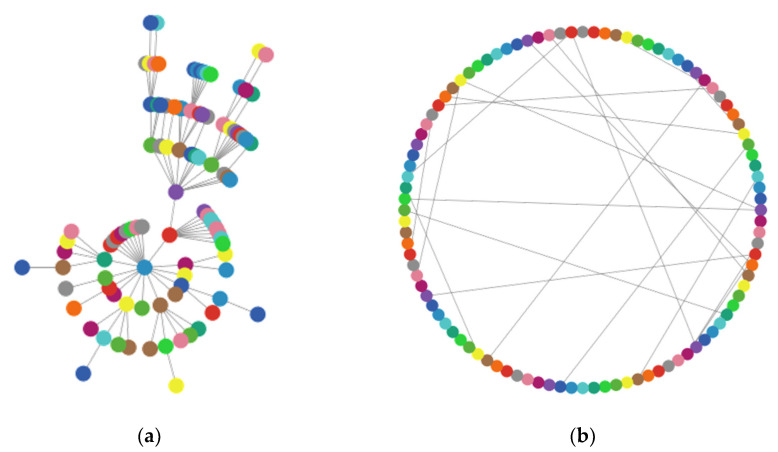
(**a**) Scale-free network; (**b**) small-world network.

The scale-free network and small-world network have been configured with 400 nodes (see Figure 6). These nodes have been distributed as follows: 400 sensor nodes and 20 random intransmissible nodes.

In the scale-free network, the graph is generated using the Barabási–Albert model. This network has the property of being “scale-free”: the distribution of degrees (i.e., the number of links for each turtle) follows a power law. The minimum degree is 1—each newly added node creates an edge with the existing nodes.

The small-world network uses Watts–Strogatz model to produce a graph with small-world properties, including short average path lengths and high clustering. The algorithm creates a ring of nodes first, where each node is connected to three nodes on either side. Then, each link is rewired with a probability of 0.05.

**Figure 7:**
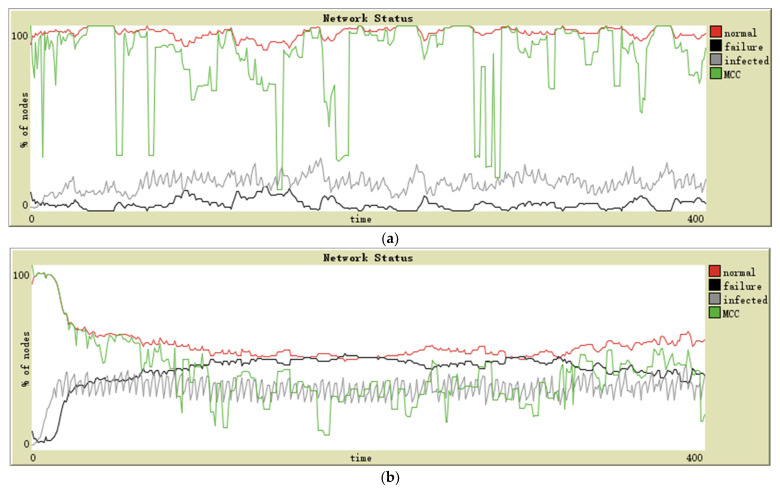
Simulation results of a (**a**) scale-free network, (**b**) small-world network.

Graphs obtained by simulation on complex networks are as follows (Figure 7a,b). The evolution of the MCC in each topology is observed.

To demonstrate the significance of malware propagation considered in WSN reliability modeling, we use the reliability model proposed in [26] for comparison, which uses the dual-mode (on and off) SN model. To make a fair comparison, we use our proposed complex network topology. The dual-mode SN model assumes that a given SN is either in a fully functional or failed state. The SN does not have a negative but unfailed state of being infected. Malware propagation is not considered, and the number of maintenance resources is reduced to maintain the fairness of comparison because the impact of malware on reliability is eliminated.

## 5. Discussion

An SEIRS-F agent-based model is introduced to simulate the component failure and malware propagation on wireless sensor networks. Agent and transition rules constitute this model. Each agent is divided into the following categories: susceptible, infected, exposed, recovered, and failed.

The model is simulated in three different scenarios, corresponding to the three typical working environments of WSN. In each case, different characteristics are established to simulate the deployment environment. The parameter settings of the three network topologies are consistent in different scenarios. The simulation results are analyzed according to each scene and each topology.

### 5.1. Results per Scenario

The first scenario of simulation corresponds to the military application environment. In this type of network, the nodes’ reliability and security tend to be high. Additionally, the attacker’s technical strength is the strongest, and often regardless of cost; for these reasons, the infection intensity in these networks is high. As shown in Figure 3a, the MCC maintains consistency with the normal state for a large proportion of the time and is 0 in the rest time. Aggregation and separation of two curves mean that these nodes can maintain the connection relationship as long as the central node functions correctly, but it is all over if the central node fails. In the lattice-2d topology, the number of infected sensors increased slowly in the early stage. The stability and consistency of the number of normal nodes and MCC are maintained after the spread of malware. The random topology shows a slightly worse MCC than that of lattice-2d; in general, both can maintain the connectivity of the remaining normal nodes when a considerable number of sensors fail.

The simulation environment has been performed in the industry in the second scenario. In these networks, the sensors may have high reliability and medium security level; attackers are willing to attack industrial infrastructure to demand ransom from businesses. In Figure 4, it is observed that the last two topologies maintain acceptable MCC performance. However, different from the military scenario, the MCC performance of lattice-2d topology is worse than that of random topology, which shows that after the proportion of failure nodes in all nodes rises to a certain extent, the lattice-2d network may be “divided” by failure nodes. In contrast, random topology with more global connectivity will not be so split. Not surprisingly, the problem with star topology is the same as in the previous scenario—dramatic mutation.

The third scenario of simulation has been designed as an environment of daily activities and Smart Home. In these networks, the nodes’ reliability and security tend to be low. The attacker also has no interest in attacking individuals. In this case, all the performances of the three topologies are unacceptable, especially star topology. Only random topology networks have been able to maintain a low-quality MCC (see Figure 5).

### 5.2. Results per Topology

In star topology, almost all nodes communicate through the central node. In this case, the central node controls the infection—other nodes can only contact no more than one infectious source. However, when the central sensor node has been infected and failed, The spread of malware will be accelerated, and MCC will plummet to 0. Therefore, controlling the infection and failure of the central sensor is an effective way to improve the robustness.

The lattice-2d topology presents a 2D lattice network; the average degree of nodes is about 4. It can maintain a stable connection between nodes in case of a node failure and will control the spread of malware to a certain extent in the earlier stage—each node is only connected to two to four nearby nodes. However, it will spread and maintain a certain number of infected nodes eventually. Moreover, if the number of failed nodes exceeds a particular proportion, the MCC of lattice-2d topology will deteriorate more than random topology.

In general, random topology has the best MCC performance. In this network type, each node has a connection probability (between 0 and 1) of being connected to each other node, which means that all nodes are able to communicate with distant ones, and the connection between nodes is homogeneous. In this case, the network can always maintain a certain degree of connectivity, even if a considerable number of nodes fail.

The topology that performs the best is the random topology. We try a simulation that only considers the failure caused by subsystem failure and external physical damage; then, the performance of random topology is also excellent. However, with the integration of malware propagation factors, the global spread of malware will drastically reduce the reliability of the nodes, thereby reducing the functionality of the network. Furthermore, a completely uniform connectivity network does not exist in reality; ignoring the distance between nodes to establish an average connection is unacceptable in cost. The star topology can be considered to have the same MCC and number of normal nodes—unless the central node fails, it will lead to the collapse of network performance. Finally, the lattice-2d topology has a performance in the middle of the other two.

### 5.3. Results of Complex Networks

The class of scale-free networks has power-law degree distributions. In this case, a small number of nodes with high connectivity become critical connection points. They can keep the infection within a particular proportion, and then the number of failed nodes is controlled. Moreover, due to their crucial role in maintaining network connectivity, their failure will divide the normal nodes into different connected components. This feature leads to the difference between the MCC and the number of normal nodes—it is easy to observe that the proportion of normal nodes is always high, but MCC often fluctuates wildly (see Figure 7a).

In a small world, the underlying lattice structure of the model produces a locally clustered network, while the randomly rewired links dramatically reduce the average path lengths. In this case, the spread of infection rapidly expands and then leads to a large number of node failures. Because the node across the clusters is the critical connective node for its cluster, its failure will also cause the cluster to be separated from the network, significantly reducing the MCC (see Figure 7b).

**Figure 8:**
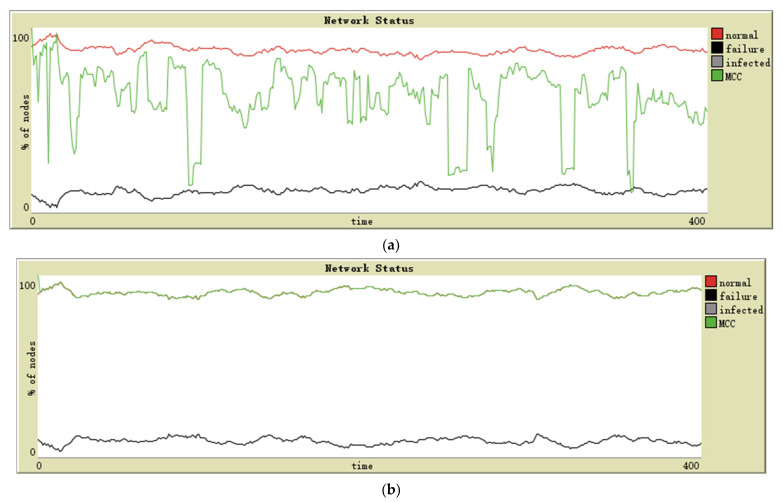
Simulation results of (**a**) scale-free network and (**b**) small-world network; malware propagation was not considered.

As can be observed from Figure 8, the MCC performance of the scale-free network in Figure 8a is somewhat different compared to Figure 7a, but it can be seen that if the number of maintenance resources is slightly increased so that the number of failed nodes in Figure 7a and Figure 8a is approximately equal, then the MCC performance should also be approximately the same in both cases. However, Figure 8b shows a significant difference compared to Figure 7b, where malware can propagate rapidly on a small-world network, massively increasing the number of failed nodes and crippling MCC performance. Once the impact of malware is eliminated, the number of failed nodes in the network remains low and the small-world network can maintain connectivity between normal nodes very well.

## 6. Conclusions and Future Work

In this paper, an ABM for connectivity robustness was introduced, integrating component failure with the malware propagation model. ABM uses MCC as a measure of connectivity robustness. Furthermore, the ABM models cascading failures between malware propagation and components, crossing the information domain and the physical domain. Finally, we used NetLogo to evaluate the connectivity robustness of different scenarios and network topologies, and the experimental results were discussed.

Below are the main conclusions drawn from the results: (1) The presented model can be used to provide evaluation results of the connectivity robustness of WSNs. (2) The network with a higher average degree has better connectivity robustness with only the component failure considered. However, considering the influence of malware propagation, high connectivity WSNs may not be robust. The trade-offs of high connectivity for rerouting and network topology for malware propagation resistance need further consideration. (3) The interactions between the physical domain and information domain have apparent impacts on the robustness performance of WSNs, which should be appropriately taken into account in the planning and designing of WSNs. (4) The visualization of the results is easy to understand, and the MCC can be analyzed in each step of time t.

In future work, the coupling relationship between the physical domain and the information domain should not be limited to one-to-one. The one-to-many coupling in the actual situation should also be taken into account. The new coupling relationship will also affect the modeling of cascading failure. Finally, a new optimization method to obtain the optimal network topology in different scenarios is needed.

## Figures and Tables

**Table 1:** Factors with their characteristics.

Type of Factors	Characteristics	Values
Sensors	Reliability state of sensor	Normal state
		Failure state
	Reliability level	Low
		Medium
		High
	Security level	Low
		Medium
		High
	Transmission capability	True
		False
	Battery power	Low
		Medium
		High
Malware	Infection intensity	Low
		Medium
		High
	Target	Software function
		Subsystem hardware
		Battery power
Network topology	Type of topologies	Star topology
		Lattice-2d topology
		Random topology
Environment	Risk of physical attack	Low
		Medium
		High
	Risk of malware attack	Low
		Medium
		High
Maintenance	Resource	Low
		Medium
		High
	Maintenance behavior	Repair
		Recharge
		Replacement
	Maintenance cycle	Low
		Medium
		High

## Data Availability

The simulation files can be found at https://github.com/pauxavi/cyber-physical-social-system-sims (accessed on 7 July 2021).

## Appendix A

The content of Appendix A is the model parameters corresponding to each scenario.

**Table A1:** Scenario 1 with characteristics and values.

Type of Factors	Characteristics	Values
Sensors	Reliability level	High
	Security level	High
	Battery power	High
Malware	Infection intensity	High
	Target	Software function
		Subsystem hardware
		Battery power
Environment	Risk of physical attack	High
	Risk of malware attack	High
Maintenance	Resource	High
	Maintenance behavior	Replacement
	Maintenance cycle	Low

**Table A2:** Scenario 1 with simulation parameters and values.

Simulation Parameter	Related Characteristics	Values
Spread chance	Security level	20%
	Infection intensity	
Initial infection number	Risk of malware attack	5
Initial failure number	Risk of physical attack	20
Failure rate	Reliability level	5%
Immunity duration	Security level	6
	Infection intensity	
Recovery chance	Security level	30%
	Infection intensity	
Resistance chance	Security level	30%
	Infection intensity	
Battery power	Battery power	30
Maintenance resource	Maintenance resource	30
Maintenance behavior	Maintenance behavior	Replacement (2 resource consumption)
Maintenance cycle	Maintenance cycle	4

**Table A3:** Scenario 1 with simulation parameters and values (complex network).

Simulation Parameter	Related Characteristics	Values
Spread chance	Security level	20%
	Infection intensity	
Initial infection number	Risk of malware attack	10
Initial failure number	Risk of physical attack	40
Failure rate	Reliability level	5%
Immunity duration	Security level	6
	Infection intensity	
Recovery chance	Security level	30%
	Infection intensity	
Resistance chance	Security level	30%
	Infection intensity	
Battery power	Battery power	30
Maintenance resource	Maintenance resource	80
Maintenance behavior	Maintenance behavior	Replacement (2 resource consumption)
Maintenance cycle	Maintenance cycle	4

**Table A4:** Scenario 2 with characteristics and values.

Type of Factors	Characteristics	Values
Sensors	Reliability level	Medium
	Security level	Medium
	Battery power	Medium
Malware	Infection intensity	High
	Target	Software function
		Battery power
Environment	Risk of physical attack	Low
	Risk of malware attack	Medium
Maintenance	Resource	High
	Maintenance behavior	Recharge
		Repair
	Maintenance cycle	Medium

**Table A5:** Scenario 2 with simulation parameters and values.

Simulation Parameter	Related Characteristics	Values
Spread chance	Security level	30%
	Infection intensity	
Initial infection number	Risk of malware attack	3
Initial failure number	Risk of physical attack	5
Failure rate	Reliability level	10%
Immunity duration	Security level	4
	Infection intensity	
Recovery chance	Security level	20%
	Infection intensity	
Resistance chance	Security level	20%
	Infection intensity	
Battery power	Battery power	15
Maintenance resource	Maintenance resource	25
Maintenance behavior	Maintenance behavior	Recharge (0.5 resource consumption)
		Repair (1 resource consumption)
Maintenance cycle	Maintenance cycle	2

**Table A6:** Scenario 3 with characteristics and values.

Type of Factors	Characteristics	Values
Sensors	Reliability level	Low
	Security level	Low
	Battery power	Low
Malware	Infection intensity	Low
	Target	Software function
		Battery power
Environment	Risk of physical attack	Low
	Risk of malware attack	Low
Maintenance	Resource	Low
	Maintenance behavior	Recharge
		Repair
	Maintenance cycle	High

**Table A7:** Scenario 3 with simulation parameters and values.

Simulation Parameter	Related Characteristics	Values
Spread chance	Security level	25%
	Infection intensity	
Initial infection number	Risk of malware attack	1
Initial failure number	Risk of physical attack	0
Failure rate	Reliability level	15%
Immunity duration	Security level	6
	Infection intensity	
Recovery chance	Security level	5%
	Infection intensity	
Resistance chance	Security level	5%
	Infection intensity	
Battery power	Battery power	10
Maintenance resource	Maintenance resource	20
Maintenance behavior	Maintenance behavior	Recharge (0.5 resource consumption)
		Repair (1 resource consumption)
Maintenance cycle	Maintenance cycle	1
